# Expression profile of Wilms Tumor 1 (WT1) isoforms in undifferentiated and all-trans retinoic acid differentiated neuroblastoma cells

**DOI:** 10.18632/genesandcancer.94

**Published:** 2016-01

**Authors:** Grazia Maugeri, Agata Grazia D'Amico, Daniela Maria Rasà, Rita Reitano, Salvatore Saccone, Concetta Federico, Rosalba Parenti, Gaetano Magro, Velia D'Agata

**Affiliations:** ^1^ Sections of Human Anatomy and Histology, Department of Biomedical and Biotechnological Sciences, University of Catania, Catania, Italy; ^2^ San Raffaele Telematic University of Rome, Rome, Italy; ^3^ Section of Animal Biology, Department of Biological, Geological and Environmental Sciences, University of Catania, Catania, Italy; ^4^ Section of Physiology, Department of Biomedical and Biotechnological Sciences, University of Catania, Catania, Italy; ^5^ Section of Anatomic Pathology, Department of Medical and Surgical Sciences and Advanced Technologies, G.F. Ingrassia, Azienda Ospedaliero-Universitaria “Policlinico-Vittorio Emanuele”, University of Catania, Catania, Italy

**Keywords:** Wilm's tumor 1 gene, tumor suppressor gene, alternative splice variants, WT1 isoforms, neuroblastoma, PI3K/Akt and MAPK/ERK signalling pathway

## Abstract

Wilms tumor 1 gene (WT1) is a tumor suppressor gene originally identified in nephroblastoma. It is also expressed in neuroblastoma which represents the most aggressive extracranial pediatric tumor. Many evidences have shown that neuroblastoma may undergo maturation, by transforming itself in a more differentiated tumors such as ganglioneuroblastoma and ganglioneuroma, or progressing into a highly aggressive metastatic malignancy. To date, 13 WT1 mRNA alternative splice variants have been identified. However, most of the studies have focused their attention only on isoform of ∼49 kDa. In the present study, it has been investigated the expression pattern of WT1 isoforms in an *in vitro* model of neuroblastoma consisting in undifferentiated or all-trans retinoic acid (RA) differentiated cells. These latter representing the less malignant phenotype of this tumor. Results have demonstrated that WT1.1-WT1.5, WT1.6-WT1.9, WT1.10 WT1.11-WT1.12 and WT1.13 isoforms are expressed in both groups of cells, but their levels are significantly increased after RA treatment. These data have also been confirmed by immunofluorescence analysis. Moreover, the inhibition of PI3K/Akt and MAPK/ERK, that represent two signalling pathway specifically involved in NB differentiation, induces an overexpression of WT1 isoforms. These data suggest that WT1 isoforms might be involved in differentiation of neuroblastic into mature ganglion cells.

## INTRODUCTION

Wilms tumor 1 gene (WT1) has been firstly identified as a tumor suppressor gene in renal nephroblastoma, also known as Wilms tumor [1, 2]. In normal tissues, WT1 plays a regulatory role in cell growth and development [3]. In embryonal and adult tissues, it is prevalently expressed in the urogenital system, central nervous system and hematopoietic organs, including the bone marrow and lymphonodes [4, 5]. Recently, WT1 has also been immunolocalized in the cytoplasm of several developing tissues, such as blood vessels (endothelial cells), skeletal muscle, radial glia and neuroblasts of the sympathetic peripheral nervous system [6-9]. WT1 is located on chromosome 11p13 and encodes a complex pattern of mRNA species [10]. A combination of alternative splicing, alternative translation start sites and RNA editing produces at least 36 distinct isoforms [10-13]. In mammals, through the mechanism of alternative splicing many transcripts are generated. To date, UniProt databank (http://www.uniprot.org) lists 13 mRNA variants encoding 13 proteins. Other mechanisms, such as alternative start codons and RNA editing, produce other isoforms. A previous paper has demonstrated that translation initiation at an upstream, in-frame CUG codon, and in-frame AUG codon downstream, generates, at least, eight WT1 proteins [3]. The existence of these different isoforms may explain the different and occasionally opposite functions assigned exclusively to full length WT1 protein [4-13]. Many studies have been focused on isoforms generated by alternative splicing of exons 5 and 9. In particular, exon 5 encodes 17 amino acids which are localized between the Pro/Glu-rich N-terminus and Zn finger domain. Instead, exon 9 results in inclusion of lysine, threonine and serine (KTS), between the third and fourth Zn finger. The mRNA transcript including both exons is predominantly expressed either in human and mouse, whereas less common is the transcript missing all of them [3].

Although WT1 has been originally recognized as a tumor suppressor gene, some studies have demonstrated that its expression increased during development and progression of different human cancers, such as hematological malignancies and solid tumors [5, 14-17].

Neuroblastoma, the most undifferentiated form of neuroblastic tumors [18], may regress spontaneously, undergo maturation into ganglioneuroblastoma or ganglioneuroma, or progress to highly aggressive metastatic disease with a poor overall survival rate [19]. Neuroblatoma is traditionally considered as a WT1-negative tumor, but a few studies have shown a variable (often focal and weak) nuclear and/or cytoplasmic expression of WT1 in this tumor [17, 20-23]. WT1 seems to act as tumor suppressor gene in NB, by inducing the maturation of this tumor [23]. Indeed, it has been reported to participate in neuronal cell differentiation [24] and [25]. To date, most of the studies have focused their attention exclusively on isoform of ∼49 kDa molecular weight. However, data regarding the expression profile of WT1 isoforms in neuroblastoma cells have not yet been described. In the present study, therefore, it has been analyzed for the first time the expression pattern of WT1 proteins in undifferentiated and all-trans retinoic acid (RA) differentiated neuroblastoma cells in order to evaluate their involvement in tumor malignancy.

Our results have showed that WT1.1-WT1.5, WT1.6-WT1.9, WT1.10, WT1.11-WT1.12 and WT1.13 isoforms are expressed both in untreated (undifferentiated) and treated (RA) neuroblastoma cells by using two antibodies recognizing different domains of canonical protein, and their expression is significantly increased in the latter cells, suggesting that WT1 isoforms are inversely related to oncogenicity of NB. Furthermore, the inhibition of PI3K/Akt and MAPK/ERK signalling pathways, that are specifically involved in differentiation of NB, induces as direct response, an overexpression of all WT1 isoforms. Therefore, they could promote directly the trans-differentiation of this tumor. Further studies are needed to structurally and functionally characterize each of these WT1 isoforms.

## RESULTS

### Expression profile of WT1 isoforms in undifferentiated and differentiated neuroblastoma cells

**Figure 1:**
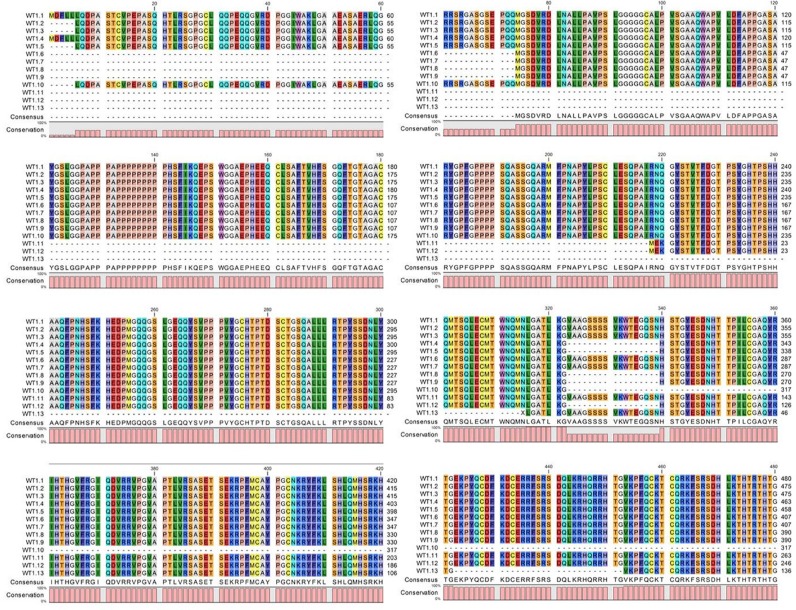
Alignment of WT1 isoforms Multiple alignment of WT1 isoforms showing the matching regions to canonical sequence WT1.6.

**Figure 2:**
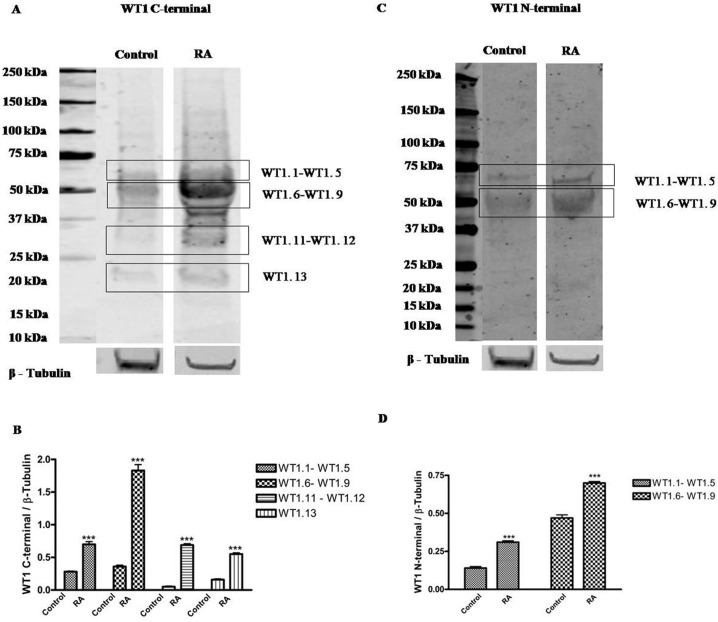
Expression profile of WT1 isoforms in undifferentiated and RA differentiated neuroblastoma cells Representative immunoblot of WT1 isoforms expression on lysate from SH-SY5Y cells cultured in growth medium (Control) or added with 10 μM *all-trans* retinoic acid (RA), detected by WT1 C-terminal (A) and WT1 N-terminal antibody (C). Blot shows the results of three independent experiments. The boxes frame signals detected from each isoform with relative ID Code, as listed in Table 1. (B-D) Relative density of each band was quantified using ImageJ software. Each signal was normalized on correspondent β-tubulin signal. Data are expressed as mean ± S.E.M. ***p<0,001 vs Control, as determined by unpaired two-tailed Student t-test.

To date, thirteen different alternative splice variants have been reported in UniProt databank (http://www.uniprot.org). These isoforms have been listed and, for simplicity, renamed with an ID code in Table 1. For each variant, it has been also reported the NCBI and ENSEMBL code (if available), the amino acidic length, the corresponding molecular weight and predicted isoelectric point obtained through ExPASy (Bioinformatics Resource Portal). For convenience, in table 1, isoforms have been organized in decreasing order based on their MW. Furthermore, to better explain which portions of each isoform corresponds to the protein WT1.6, here chosen as ‘canonical’ sequence, the alignment of predicted amino acid sequences has been performed as shown in Figure 1. To establish which WT1 isoforms are expressed in human undifferentiated and differentiated SH-SY5Y neuroblastoma cells, it has performed western blot analysis, by using two antibodies, that recognize different domains of WT1.6 isoform. More specifically, it has used WT1 (C-19) antibody that maps at the C-terminus of WT1 of human origin; and WT1 (Clone 6F-H2) antibody, that reacts with all isoforms of the full-length WT1 and also identifies WT1 lacking exon 2-encoded amino acids. When the amino acids sequence recognized by each antibody perfectly match with the sequence of the full length protein, it is very likely to get a signal by western blot and this is indicated in the table 1 by ‘Yes’. If the antibody recognizes at least 8 consecutive amino acids on the protein, this is indicated in the table 1 by “May be”. Instead, if the antibody recognizes less than 8 consecutive amino acids, it could rule out the possibility to visualize a signal on immunoblot and this is indicated in the table by ‘No’. As shown in Figure 2A, control and differentiated cells (RA) express different levels of WT1.1-WT1.5, WT1.6-WT1.9, WT1.11-WT1.12 and WT1.13 isoforms, corresponding to bands of ∼55 kDa, ∼50 kDa, ∼33 kDa and ∼20 kDa molecular weight, respectively, by using WT1 C-terminal antibody.

**Table 1:** WT1 isoforms recognized by WT1 C-terminal and N-terminal antibody

ID CODE	UNIPROT ID	NCBI ID	ENSEMBL ID	LENGTH aa	MW	pI	Ab C-term	Ab N-term
WT1. 1	P19544-7	/	/	522	56,88	9,10	yes	yes
WT1. 2	J3KNN9	NP_077744	ENST00000332351	517	56,26	9,16	yes	yes
WT1. 3	H0Y7K5	NP_077742	ENST00000448076	514	55,95	9,11	maybe	yes
WT1. 4	P19544-8	/	/	505	55,21	9,11	yes	yes
WT1. 5	A0A0A0MT54	NP_000369	ENST00000452863	497	54,27	9,12	maybe	yes
*WT1. 6	P19544-1	AAO61088	/	449	49,19	9,23	yes	maybe
WT1. 7	P19544-4	EAW68220	/	446	48,87	9,18	maybe	maybe
WT1. 8	P19544-3	EAW68221	/	432	47,51	9,24	yes	maybe
WT1. 9	P19544-2	/	/	429	47,19	9,19	maybe	maybe
WT1. 10	H0Y3F0	/	ENST00000379077 Nonsense mediated decay	317	33,04	5,76	NO	yes
WT1. 11	P19544-6	NP_001185480	ENST00000379079	302	34,45	9,53	maybe	NO
WT1. 12	P19544-9	NP_001185481	ENST00000530998	288	33,09	9,59	yes	NO
WT1. 13	H0YED9	/	ENST00000527882	178	20,20	10,25	yes	NO

* WT1.6 is the ‘canonical’ sequence

Yes: perfect match between predicted protein sequence and antibody epitope.

No: matching between predicted protein sequence and antibody epitope is less than 8 consecutive amino acids. May be: partial match between predicted protein sequence and antibody epitope.

Only two bands of ∼55 kDa and ∼50 kDa, corresponding to WT1.1-WT1.5 and WT1.6-WT1.9 isoforms, are observed in the blot by using WT1 N-terminal antibody (Figure 2C), even if as predicted in table 1, WT1.10 isoform was expected. This result might depend on the different affinity of antibody toward the various isoforms.

WT1 isoforms expression is significantly increased in RA treated cells as compared to control, by using both antibodies (Figure 1B-D) (***p<0,001 vs Control, as determined by unpaired two-tailed Student t-test).

**Figure 3:**
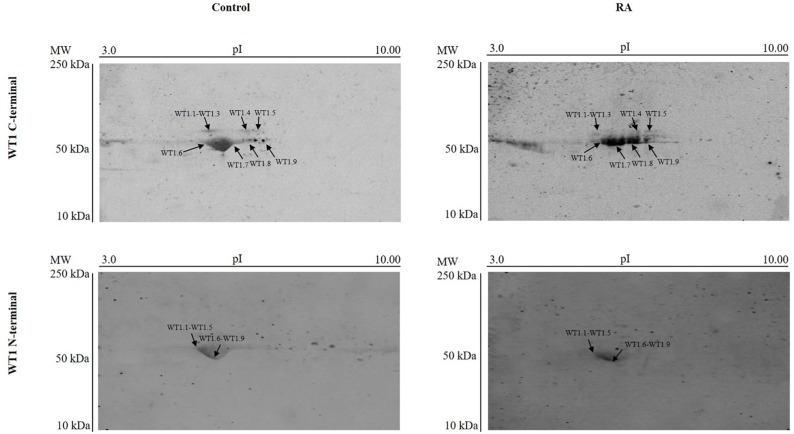
Representative 2D gel immunoblots of WT1 protein isoforms expressed in undifferentiated and RA differentiated neuroblastoma cells The images are representative of two-dimensional electrophoresis blots showing WT1 signals detected on lysate from SH-SY5Y cells cultured in growth medium (Control) or added with 10 μM *all-trans* retinoic acid (RA), detected by WT1 C-terminal and WT1 N-terminal antibody. The blots are representative of three independent experiments.

To discriminate among these isoforms, it has been carried out 2D SDS-PAGE analysis. The blots show the presence of some isoforms with a molecular weight of ∼56-50 kDa by using WT1 C-terminal antibody. Instead, a single spot of ∼50 kDa is identified by using WT1 N-terminal antibody (Figure 3). Probably, only these signals have been detected, since they represent the isoforms more expressed, as visualized by western blot analysis. However, the PI point does not match to that predicted on table 1. It cannot rule out that the dissimilarity may be due to post-translation modifications of these isoforms not considered.

**Figure 4:**
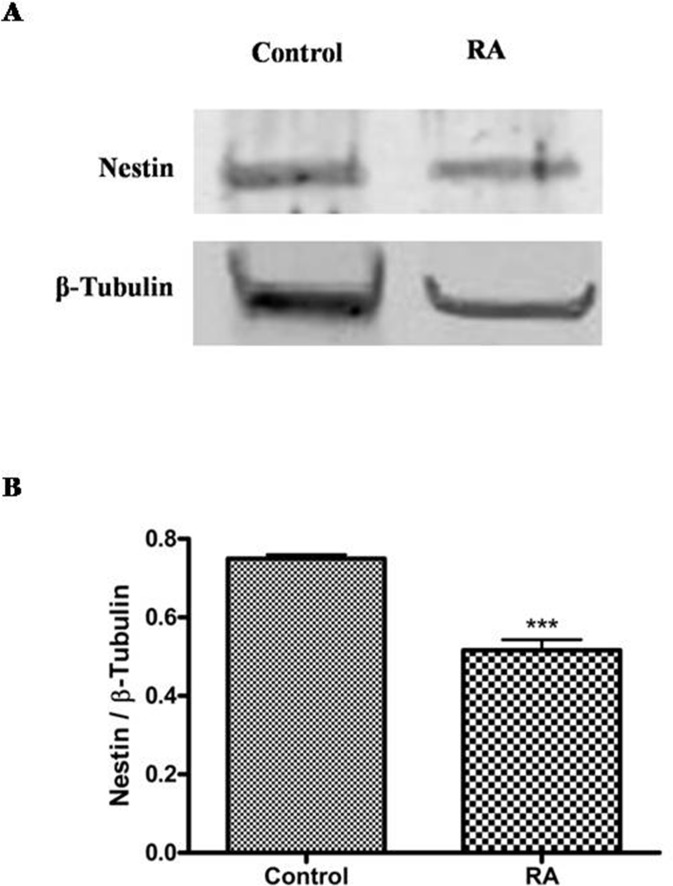
Nestin expression in undifferentiated and RA differentiated neuroblastoma cells (A) Representative immunoblots of nestin expression on lysate from SH-SY5Y cells cultured in growth medium (Control) or added with 10 μM *all-trans* retinoic acid (RA). Blot shows the results of three independent experiments. (B) Relative density of each band was quantified using ImageJ software. Each signal was normalized on correspondent β-tubulin signal. Data are expressed as mean ± S.E.M. ***p<0,001 vs Control as determined by unpaired two-tailed Student t-test.

To correlate these results to tumor aggressiveness, it has been analyzed the expression of nestin, a neuroectodermal stem cell marker, in control and RA treated cells by western blot analysis. As shown in Figure 4A, cell differentiation significantly reduces the expression of nestin in RA group as compared to control (***p<0,001 vs Control, as determined by unpaired two-tailed Student t-test) (Figure 4B). This data could suggest that WT1 isoforms and nestin expressions are inversely related in undifferentiated and RA treated cells.

### WT1 isoforms involvement in differentiation of neuroblastoma cells

**Figure 5:**
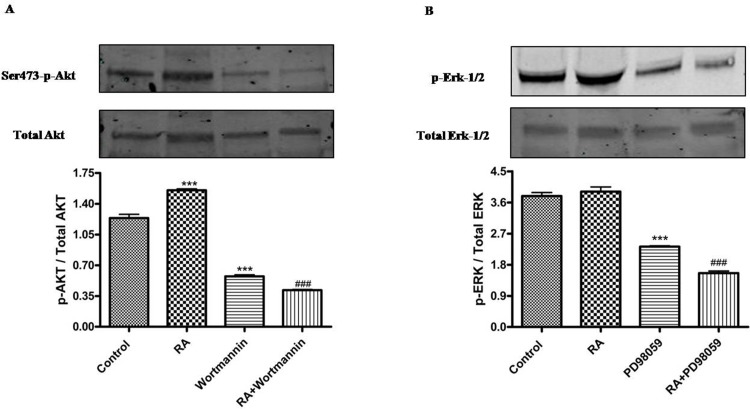
Phosphorylation of AKT and ERK1/2 in undifferentiated and RA differentiated neuroblastoma cells (A) Phosphorylation of Ser473Akt in SH-SY5Y cells cultured in growth medium (Control) or added with 10 μM *all-trans* retinoic acid (RA), or with 10 μM Wortmannin (Wortmannin), or with 10 μM *all-trans* retinoic acid and 10 μM Wortmannin (RA+Wortmannin), as determined by Western blots. Relative density of each band was quantified using ImageJ software. Each signal of phosphorylated protein was normalized to total protein expression. Data are expressed as mean ± S.E.M. ***p<0,001 vs Control; ###p<0,001 vs RA as determined by one-way ANOVA followed by the Tukey post hoc test. (B) Phosphorylation of Erk1/2 in SH-SY5Y cells cultured in growth medium (Control) or added with 10 μM *all-trans* retinoic acid (RA), or added with 50 μM PD98059, or with 10 μM *all-trans* retinoic acid and 50 μM PD98059 (RA+PD98059), as determined by Western blots. Relative density of each band was quantified using ImageJ software. Each signal of phosphorylated protein was normalized to total protein expression. Data are expressed as mean ± S.E.M. ***p<0,001 vs Control; ###p<0,001 vs RA as determined by one-way ANOVA followed by the Tukey post hoc test.

To characterize the functional role of WT1 isoforms in trans-differentiation of NB cells, their expression profile has been correlated to two different signaling pathways, critically involved in this malignancy, namely, phosphoinositide 3 kinase (PI3K) /Akt and mammalian mitogen activated protein kinase/Erk kinase (MAPK/ERK) signaling pathways. As shown in Figure 5, in RA treated cells is significantly increased AKT phosphorylation at Ser473, as compared to controls (***p<0,001 vs Control as determined by one-way ANOVA followed by the Tukey post hoc test).

Furthermore, phosphorylation/activation of Erk-1/2 is also increased in differentiated as compared to undifferentiated cells, although this result is not statistically significant.

To confirm the involvement of these pathways in SH-SY5Y cell differentiation, PI3K pathway inhibitor, Wortmannin, and MAPK/ERK pathway inhibitor, PD98059, have been added in differentiated and undifferentiated cells. Both Wortmannin or PD98059 treatments have significantly reduced AKT or ERK phosphorylation, respectively, as compared to control (***p<0,001 vs Control; ###p<0,001 vs RA as determined by one-way ANOVA followed by the Tukey post hoc test) (Figure 5).

**Figure 6:**
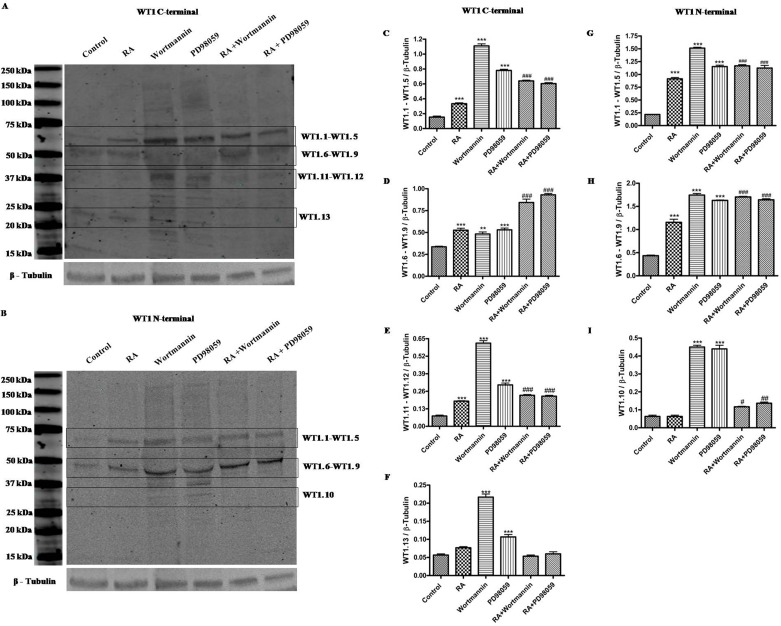
Expression profile of WT1 isoforms after inhibition of the phosphoinositide 3-kinase (PI3K)/Akt signaling pathway or of the mammalian mitogen activated protein kinase/Erk kinase (MAPPK or MEK) family Representative immunoblot of WT1 isoforms expression, detected by WT1 C-terminal (A) and WT1 N-terminal (B) antibody, on lysate from SH-SY5Y cells cultured in growth medium (Control), or added with 10 μM *all-trans* retinoic acid (RA), or with 10 μM Wortmannin (Wortmannin), or with 50 μM PD98059 (PD98059), or with 10 μM *all-trans* retinoic acid and 10 μM Wortmannin (RA+Wortmannin), or with 10 μM *all-trans* retinoic acid and 50 μM PD98059 (RA+PD98059). (C-G) Relative density of each band was quantified using ImageJ software. Each signal was normalized on correspondent β-tubulin signal. Data are expressed as mean ± S.E.M. ***p<0,001 vs Control; ###p<0,001 vs RA as determined by one-way ANOVA followed by the Tukey post hoc test.

To correlate the expression profile of WT1 isoforms to activation of both PI3K/Akt or MAPK/ERK signaling pathways, we have analyzed their expression in SH-SY5Y cells before and after RA differentiation following Wortmannin or PD98059 treatments. As shown in Figure 6, inhibition of these signaling pathways has increased the expression levels of WT1 isoforms in all experimental groups. Furthermore, surprisingly, a band of ∼33 kDa, corresponding to WT1.10 isoform has been detected on blot only after inhibition of these signaling pathways in undifferentiated cells (***p<0,001 vs Control; ###p<0,001 vs RA, as determined by one-way ANOVA followed by the Tukey post hoc test) (Figure 6I)”. As expected in table 1, this isoform is detected on blot only by using the N-terminal antibody.

### Immunolocalization of WT1 isoforms and Nestin in undifferentiated and RA differentiated neuroblastoma cells

To investigate WT1 isoforms distribution in SH-SY5Y cells, before and after RA differentiation, we have performed immunofluorescence analysis by using confocal laser scanning microscopy. The results have been correlated to cell's malignancy by evaluating their co-localization with nestin, a marker associated to tumor aggressiveness.

**Figure 7:**
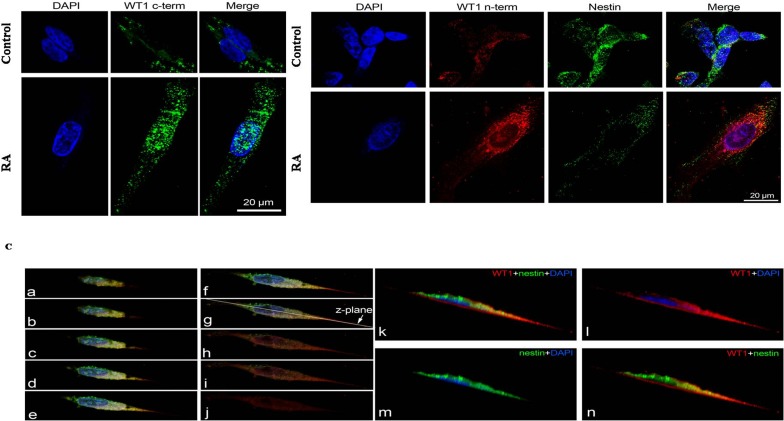
Immunolocalization of WT1 isoforms and Nestin in undifferentiated and RA differentiated neuroblastoma cells (A) Representative photomicrographs show total WT1 isoforms detected by WT1 C-terminal antibody (green) in SH-SY5Y cells. Nuclei were stained with DAPI (blue). (B) Representative photomicrographs show immunolocalization of total WT1 isoforms detected by WT1 N-terminal antibody (red) and Nestin protein (green) in SH-SY5Y cells. Nuclei were stained with DAPI (blue). (C) Immunolocalization of total WT1 isoforms detected by WT1 N-terminal antibody (red) and Nestin protein (green). a to j: serial z-sections (steps of 0.5 μm) obtained by confocal laser scanning microscope. k to n: image along the z-plane indicated in the panel g. Cells were cultured in growth medium (Control) or added with 10 μM all-trans retinoic acid (RA). Photomicrographs are representative results of fields taken randomly from slide and scanned by confocal laser scanning microscopy (CLSM; Zeiss LSM700).

To date, there are not commercially available antibodies which allow to discriminate among the WT1 isoforms. This limits the immunolocalization analysis because, the detected immunofluorescence, represents the combination of signals generated by the isoforms expressed in each cells. However, the data obtained have confirmed the results of western blot. In control cells, a weak cytoplasmic immunoreactivity of WT1 has been observed, by using both antibodies (Figure 7 A-B), instead, in these cells, nestin is highly expressed in cytoplasm and in the perinuclear region (Figure 7 B-C). In contrast, RA treatment enhances WT1 isoforms and reduces nestin expression, respectively. In particular, WT1 is highly expressed both in cytoplasm and nucleus (Figure 7 A-B-C), whereas nestin is weakly expressed only in cytoplasm (Figure 7 B-C).

## DISCUSSION

Abnormalities in WT1 gene, have been associated with the pathogenesis of Wilms tumor, Denys-Drash syndrome, and Frasier syndrome [10, 26, 27]. WT1 seems play different functions: it induces apoptosis, inhibits cell growth, and suppresses tumorigenicity both *in vivo* and *in vitro* models [28-34]. Furthermore, in tumors with advanced clinical stage and poor prognosis, high levels of WT1 mRNA have been described. Our study is mainly focused on WT1 role in NB which represents the most common and malignant tumor of early childhood [11]. The evolution of this tumor is unpredictable, ranging from progression to a highly aggressive metastatic disease or to a spontaneous regression or maturation in ganglioneuroblastoma/ganglioneuroma [23]. A previous study has demonstrated that WT1 plays an important role in the pathogenesis of this tumor by inducing primitive neuroblastic cells to differentiate into mature less malignant ganglion cells [23]. This is consistent with the evidence that WT1 expression is greater in ganglioneuroma *versus* neuroblastoma [23].

To date, most of the studies have been focused on isoform of ∼49 kDa molecular weight. However, up to 13 WT1 alternative splice variants are currently reported on UniProt data bank. They have been listed and renamed in Table 1. By analyzing their amino acid sequence, it has been observed that these proteins have different molecular structures with potential different functions. Indeed, it has been observed that WT1 can act as a tumor suppressor gene or, in some cases, as oncogene by considering the tumor histotype [35, 36]. In our study, it has been investigated for the first time, the expression pattern of WT1 isoforms by correlating them to cellular differentiation. In this regard, it has been analyzed WT1 proteins in undifferentiated or RA differentiated neuroblastoma cells. The latter group may represent an in vitro model of ganglioneuroma, that is the most benign tumor within the spectrum of neuroblastic tumor [37]. Furthermore, in accord to this hypothesis, we have found that nestin expression levels are significantly reduced in RA differentiated as compared to undifferentiated cells, confirming the greater malignancy grade of these latter. Nestin, indeed, is a marker of multipotent neuroectodermal precursor cells, expressed in a cell-cycle-dependent manner, and its overexpression is related to tumor aggressiveness, including NB [38]. Expression analysis of WT1 isoforms, on undifferentiated and RA differentiated cells, has shown that both groups express different WT1 isoforms. In particular, WT1.1-WT1.5, WT1.6-WT1.9, WT1.11-WT1.12 and WT1.13 isoforms, have been visualized by using WT1 C-terminal antibody, instead WT1.1-WT1.5 and WT1.6-WT1.9 isoforms, have been observed in the blot by using WT1 N-terminal antibody. However, WT1 isoforms expression is significantly increased in RA treated cells compared to untreated cells. This data suggests that expression levels of WT1 isoforms are correlated to cells' differention state. This evidence has been further confirmed by 2D SDS-PAGE analysis. The blots have revealed many spots, with molecular weight ranging from ∼56 and 47 kDa, corresponding to isoforms previously detected by western blot analysis.

To investigate the direct involvement of WT1 isoforms in NB cells trans-differentiation, their expression levels have been analyzed after inhibition of the PI3K/Akt or MAPK/ERK signaling cascades. These latter are important cell survival pathways which regulate differentiation process of various cells, including NB ones [39-40]. In accord to this evidence, different studies have suggest that both PI3K and ERK1/2 play a crucial role in differentiation and survival of NB cells [37, 41]. Moreover, overexpression of PI3K is involved in neurite outgrowth and expression of some neuronal markers [42]. Our results have shown that the inhibition of these signaling pathways triggers the overexpression of WT1 isoforms. This data confirms involvement of these latter in cell differentiation, although it does not allow us to establish whom, and how, are involved in this process. In support of our hypothesis, there is the evidence that the WT1.10 isoform has been detected on blot only after inhibition of these pathways. Therefore, although it has been provided here empiric evidences, these data suggest that some WT1 isoforms might be strongly linked to NB differentiation.

These data have also been confirmed by immunofluorescence analysis. An inverse correlation in the expression levels of WT1 compared to nestin has been detected in both cell groups. Although these proteins are localized in cytoplasm, WT1 immunoreactivity is higher in differentiated as compared to undifferentiated cells, whereas nestin is highly expressed in untreated as compared to RA treated cells.

In conclusion, all these data suggest that WT1 isoforms expression is related to NB cell differentiation. This confirms previous data which have suggested that WT1 is not related to their oncogenicity and, instead, it might promote trans-differentiation of NB into a more benign ganglioneuroma/ganglioneuroblastoma.

To our opinion, despite this is a fascinating hypothesis, it does not allow to make any conclusions about the role of each WT1 isoforms in NB neither papers, to date, on this issue have been published. The identification and functional characterization of these isoforms will be further investigated in future studies by using antibody direct against specific protein domain, however, to our knowledge, there are not currently commercially available antibodies with this characteristic.

## METHODS

### Cell culture and differentiation

The human neuroblastoma cell line SH-SY5Y, obtained from American Type Culture Collection (ATCC) (Rockville, MD, USA), was grown in a mixture of 1:1 Dulbecco's modified Eagle's medium (DMEM) and Ham's F-12K Nutrient Medium supplemented with 10% of heat-inactivated fetal bovine serum (FBS), 100 U/ml penicillin, and 100-μg/ml streptomycin, and incubated at 37°C in 5% CO_2_ as previously described [43]. To analyze the expression profile of WT1 isoforms in an *in vitro* model of less aggressive neuroblastoma tumor we differentiated SH-SY5Y cells by using 10 μM *all-trans* retinoic acid in complete growth medium (RA) (Sigma Cat n. 302-79-4) for 7 days by changing the medium every two days.

### Two-dimensional gel electrophoresis (2D SDS-PAGE analysis)

For analytical 2D gels, triplicate gels from each sample were run. In total we performed a cleanup of the 400 μg proteins for each sample, as reported in Instruction Manual (ReadyPrep 2-D Cleanup Kit, Bio-Rad). These proteins were solubilized in Rehydration/Sample buffer (8 M urea, 2% CHAPS, 50 mM dithiothreitol (DTT), 0.2% (w/v) Bio-Late 3/10 ampholytes and Bromophenol trace), and applied to 11 cm Ready Strip IPG (pH 3-10). The first-dimensional separation was performed with PROTEAN i12 IEF System (Bio-Rad). Strips were placed on a plate and isoelectric focusing (IEF) was performed with step wise increasing voltage as follows: 250 V for 20 min; 8000 V for 1 h; 8,000 V for 5 h; 8,000 V for 30 min. After IEF, the strips were equilibrated with gentle shaking in two subsequent steps for 10 min each in Equilibration buffer I, a solution containing 6 M urea; 2%SDS; 0.375M Tris-HCl (pH 8.8); 20% glycerol; 2% (w/v) DTT, followed by Equilibration buffer II, a solution containing 6 M urea; 2% SDS; 0.375M Tris-HCl (pH 8.8); 20% glycerol. Strips were removed from equilibration solution and rinsed with running buffer (25mM Tris, 192 mM glycine, 0.1% w/v SDS, ph 8.3). Subsequently, placed on top of a Criterion TGX Precast gels 1 well Comb, 11 cm (Bio-Rad) and sealed in place with 1% w/v agarose in running buffer. Second dimension electrophoresis was performed using a Bio-Rad Criterion XT 4-15% Bis-tris gel (Bio-Rad) by electrophoresis and then transferred to a nitrocellulose membrane (Bio-Rad) as previously described [44].

### Western blot analysis

Western blot analysis was performed as previously described by Maugeri et al. [45-46] to determine the relative levels of WT1 isoforms. Briefly, proteins were extracted with a buffer containing 20 mM Tris (pH 7.4), 2 mM EDTA, 0.5 mM EGTA; 50 mM mercaptoethanol, 0.32 mM sucrose and a protease inhibitor cocktail (Roche Diagnostics) using a Teflon-glass homogenizer and then sonicated twice for 20 sec using an ultrasonic probe, followed by centrifugation at 10.000 g for 10 min at 4°C. Each sample containing about 40 μg of protein homogenate, was diluted in 2× Laemmli buffer (Bio-Rad, Carlsbad, CA, USA), heated at 70°C for 10 min. Proteins were separated on a Bio-Rad Criterion XT Bis-Tris 4–15% and then electro-transferred to a nitrocellulose membrane (Bio-Rad). Blots were blocked using the Odyssey Blocking Buffer (LI-COR Biosciences) and probed with appropriate antibodies: rabbit polyclonal anti-WT1 (C-19) (cat n.sc-192, Santa Cruz Biotechnology; 1:200), mouse monoclonal anti-WT1 (Clone 6F-H2) (cat M3561, Dako, 1:200), rabbit polyclonal anti-nestin (H-85) (cat n. sc-20978, Santa Cruz Biotechnology; 1:200), rabbit anti-phospho Akt (Ser473 residue) (D9E, cat no. #4060, Cell Signaling; 1:1000), rabbit anti-total Akt (C67E7, cat no. #4691, Cell Signaling; 1:1000), mouse anti-phospho Erk-1/2 (Thr202 and Tyr204 residues) (pT202/pY204.22A, cat no. sc-136521, Santa Cruz Biotechnology; 1:200), mouse anti-total Erk-1/2 (MK1, cat no. sc-135900, Santa Cruz Biotechnology; 1:200) and rabbit anti-β-tubulin (cat n.sc-9104, Santa Cruz Biotechnology; 1:500). The secondary antibody goat antirabbit IRDye 800CW(cat #926-32211, Li-Cor Biosciences), and goat antimouse IRDye 680CW (cat #926-68020D, Li-Cor Biosciences) were used at 1: 20,000 and 1:30,000, respectively. Blots were scanned with an Odyssey Infrared Imaging System (Odyssey). Densitometric analyses of western blot signals were performed at non-saturating exposures and analyzed using the ImageJ software (NIH, Bethesda, MD; available at http://rsb.info.nih.gov/ij/index.html). Values were normalized to β-tubulin, which served as loading control, as previously described by D'Amico et al. [47].

### Immunolocalization

To determine cellular distribution of WT1 and nestin proteins, immunofluorescence analysis was performed on control and RA treated cells as previously described [48-49]. They were cultured on glass cover slips, fixed in 4% paraformaldehyde in PBS (15′ at room temperature), permeabilized with 0.2% Triton X100, blocked with 0.1% BSA in PBS, and then probed with anti-WT1 (1:50) and anti-nestin (1:50) antibody. Signals were revealed with Alexa Fluor 488 goat antirabbit for 1.5 h at room temperature and shielded from light. DNA was counterstained with DAPI (#940110, Vector Laboratories). After a series of PBS and double-distilled water washes, the fixed cells were cover-slipped with Vectashield mounting medium (Vector Laboratories, Inc., Burlingame, CA, USA). Immunolocalization was analyzed by confocal laser scanning microscopy (CLSM; Zeiss LSM700). Green and blue signals were detected with laser 488nm/10mW and 405nm/5mW respectively, and using the objective “PLAN-APOCHROMAT” 63X/1,40 OIL DIC M27. Each scanning was individually digitalized by a high sensitivity PMT using the following acquisition setup: Gain master: 776; digital offset: −202; digital gain: 1.0. All acquisitions were performed with ZEN-2010 software.

### Statistical analysis

Data are reported as Mean ± S.E.M. One-way analysis of variance (ANOVA) was used to compare differences among groups, and statistical significance was assessed by the Tukey–Kramer post hoc test. The level of significance for all statistical tests was p≤0.05.
